# Web-Based Psychotherapy for Posttraumatic Stress Disorder in War-Traumatized Arab Patients: Randomized Controlled Trial

**DOI:** 10.2196/jmir.3582

**Published:** 2015-03-20

**Authors:** Christine Knaevelsrud, Janine Brand, Alfred Lange, Jeroen Ruwaard, Birgit Wagner

**Affiliations:** ^1^Department of Clinical Psychology, Freie UniversityBerlinGermany; ^2^Treatment Center for Torture VictimsBerlinGermany; ^3^Department of Clinical PsychologyUniversität AmsterdamAmsterdamNetherlands; ^4^Department of Clinical Psychology, VU UniversityAmsterdamNetherlands; ^5^Department Clinical Psychology and PsychotherapyMedical School BerlinBerlinGermany

**Keywords:** posttraumatic stress disorders, Middle East, war, violence, cognitive therapy, mental health service, Internet

## Abstract

**Background:**

In recent years, armed conflicts in the Middle East have resulted in high rates of exposure to traumatic events. Despite the increasing demand of mental health care provision, ongoing violence limits conventional approaches of mental health care provision. Internet-based interventions for posttraumatic stress disorder (PTSD) have proved feasible and effective in Western countries, but their applicability and efficacy in war and conflict regions remains unknown.

**Objective:**

This study investigated the efficacy of a cognitive behavioral Internet-based intervention for war-traumatized Arab patients, with focus on Iraq.

**Methods:**

A total of 159 individuals with PTSD participated in a parallel group randomized trial. Participants were randomly allocated by a computer-generated sequence to a treatment group (n=79) or a waiting list control group (n=80). The treatment group received 2 weekly 45-minute cognitive behavioral interventions via Internet over a 5-week period (10 sessions in total). The primary outcome was recovery from posttraumatic stress symptoms.

**Results:**

Posttraumatic stress symptoms were significantly reduced from baseline to posttreatment (intention-to-treat analysis) in the treatment group relative to the control group (F_1,157_=44.29, *P*<.001, d=0.92). Treatment effects were sustained at 3-month follow-up. Completer analysis indicated that 29 of 47 patients (62%) in the treatment group had recovered from posttraumatic stress symptoms at posttreatment (reliable change and Posttraumatic Stress Diagnostic Scale score <20) versus 1 patient (2%) in the control group (OR 74.19, 95% CI 9.93-585.8, *P*<.001) indicating that the chance of recovering was 74.19 times higher in the treatment than in the control group.

**Conclusions:**

The results indicate, even in unstable and insecure settings with ongoing exposure to human rights violations through war and dictatorships, people with posttraumatic stress symptoms benefit from a cognitive behavioral treatment provided entirely through the Internet. This method of delivery could improve patients’ access to humanitarian aid in the form of e-mental health services.

**Trial Registration:**

Australian New Zealand Clinical Trial Registry, ACTRN12611001019998; https://www.anzctr.org.au/Trial/Registration/TrialReview.aspx?id=347505 (Archived by WebCite at http://www.webcitation.org/6Wto4HCdH).

## Introduction

In recent decades, war and human rights violations in the Middle East have led to high rates of exposure to traumatic events and to a correspondingly high incidence of posttraumatic stress disorder (PTSD) in the region [1-3]. Under the Ba’ath regime, for example, Iraqis were subjected to widespread, severe human rights abuses, including torture, killings, disappearances, beatings, kidnappings, forced amputation, and rape. With the invasion of Iraq by the US-led coalition forces in 2002, exposure to traumatizing events increased dramatically, with suicide bombers killing significantly more civilians than coalition soldiers [4]. Hick and colleagues [5] reported 1003 suicide bomb events between 2003 and 2010, causing 11% of all Iraqi civilian deaths and 26% of civilian injuries. Between 2004 and 2007, Iraqis were at the highest risk worldwide of dying in a violent conflict [6]. The escalation of violence led to an unprecedented demand for medical and psychotherapeutic support. However, half of the nation’s physicians are estimated to have fled Iraq [7]. Physicians, mental health professionals, and health care professionals in general have been frequent targets of kidnappings, bodily mutilation, and random shootings; many health professionals have been killed [8,9]. Given the security situation, most international aid organizations have left the country. At the same time, violence has spread throughout the neighboring countries. Dramatic political developments (ie, the Arab Spring) in the Middle East and North Africa have been associated with state violence and civil war has further increased the demand for additional physical and mental health support structures.

Providing medical or mental health care in regions of war and ongoing violent conflict often puts mental health professionals at great risk. Very few studies have reported on mental health care services provided for survivors of war in developing countries. Although their results have been encouraging, these approaches are available to only small numbers of people, are relatively costly, and require health professionals to be located on site [10,11].

Native-speaking health professionals who are geographically independent of their clients may, therefore, be able to provide vital psychiatric support in underserved conflict regions.

Internet-based delivery of psychotherapeutic interventions has become increasingly established in the Western world. In particular, interventions developed for patients with PTSD have been shown to produce significant reductions in PTSD symptoms and in associated psychopathology, such as depression and anxiety [12-17]. Internet-based approaches may provide a unique treatment alternative in conflict areas where there is an urgent need for psychological care that is easily accessible, independent of the location of the therapist, and relatively inexpensive.

The major aim of this study was to evaluate an online cognitive behavioral therapy (CBT) intervention for posttraumatic stress symptoms in a setting that remains highly unstable, namely Iraq. This study randomly assigned participants to either an Internet-based treatment program or a waiting list control condition. Those in the intervention group received immediate access to an Internet-based program for PTSD treatment, whereas those in the control condition had to wait several weeks before they get access to the same Internet-based program thereafter. We hypothesized that the Internet-based treatment would produce a significantly greater improvement on the outcome compared to the control condition. Additionally, participants in the treatment group were asked to take part in a 3-month follow-up measure, whereas the control group did not.

## Methods

### Participants

Participants were 159 Arabic-speaking adults (45 male, 114 female) with clinical levels of posttraumatic stress, aged from 18 to 56 years (mean 28.1, SD 7.43). In all, 48 of 159 patients (30.2%) were married and 41 of 159 participants (25.8%) completed secondary school; 99 of 159 (62%) had a university degree. Concerning the type of trauma, 63 of 159 patients (39.6%) had experienced sexual violence (war-related and sexual abuse) and 24 of 159 (15.1%) had experienced the killing of a family member or close person. Moreover, 30 of 159 participants (18.9%) reported being exposed to violence (eg, kidnapping, witnessing bomb attacks) and war or torture as their index trauma.

To be included in the study, participants had to have a history of trauma according to the *Diagnostic and Statistical Manual of Mental Disorders* (Fourth Edition; *DSM-IV*) criteria accompanied by posttraumatic stress symptoms, knowledge of Arabic, and age between 18 and 65 years. The Posttraumatic Stress Diagnostic Scale (PDS) was used to identify if patients reported the minimum number of symptoms required by *DSM-IV* for each of the symptom clusters (at least 1 intrusion, 3 avoidance, and 2 hyperarousal symptoms). Additionally the minimum score on the PDS to be included in the trial was 11 indicating moderate symptom severity. Applicants were excluded if they met 1 of the following criteria: currently receiving treatment elsewhere, substance abuse or dependence, high risk of suicide, psychotic symptoms, and low symptom severity. Symptom severity of depression was assessed by the Hopkins Symptom Checklist for depression [18] and risk of suicide by the Arabic translation of the Suicide Risk Assessment [19], a 6-item self-report questionnaire designed to capture suicidal tendencies. It consists of questions identifying suicidal plans, previous suicide attempts, and current suicidal intentions. Psychotic symptoms were assessed by the Arabic translation of the Dutch Screening Device for Psychotic Disorder [20]. Because no data are yet available from an Iraqi norm group, the Dutch norm data were used; however, due to intercultural differences, these norm data may have been too conservative.

**Figure 1:**
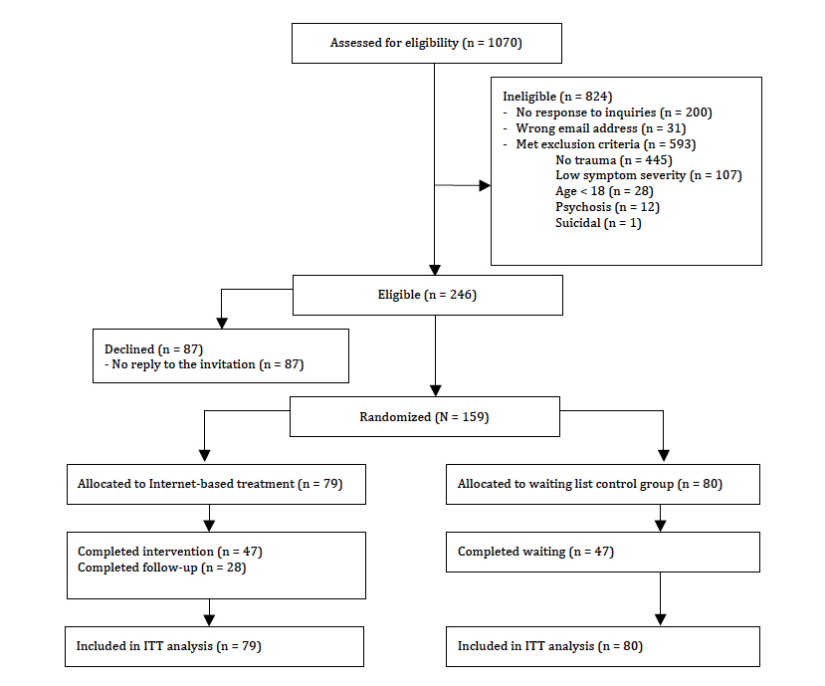
Flowchart showing progression of participants through the study.

Of the 1070 people who approached the study, 593 were excluded on the basis of our exclusion criteria (eg, outside age range, non–trauma-related difficulties, lack of Internet access). A total of 159 participants were randomly assigned to the treatment (n=79) or control condition (n=80). Participant flow is illustrated in Figure 1.

### Procedure

The study was carried out in Berlin (Treatment Center for Torture Victims, Freie University, Berlin). Recruitment for this randomized controlled trial took place from January 2009 to November 2011. Participants were recruited through radio, TV, and newspaper announcements, as well as health-related websites, specifically in Iraq. Information about the study was published regularly on a Facebook page.

**Figure 2:**
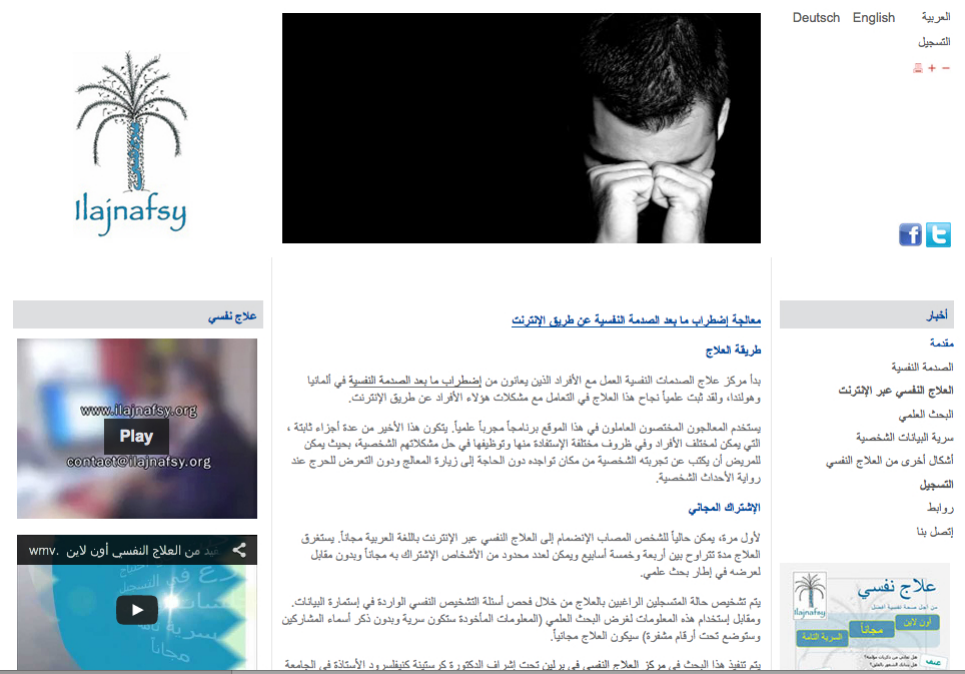
Screenshot Startpage.

The study website [21] provided general information about PTSD, online assessment, and the treatment program (Figure 2). Potential participants were informed about the study and received information about (1) posttraumatic stress reactions, (2) the study and its inclusion and exclusion criteria, (3) the Internet-based treatment, and (4) other treatment alternatives. A detailed description of the 3 treatment modules and the text-based form of the intervention was also given to the participants along with the patient information. Because the pilot study revealed that some patients had doubts about the neutrality of the website and treatment offered [22], participants were explicitly informed that all patient data and texts would be protected by rigorous security measures.

Potential patients logged in and completed the screening questionnaires online (1070 screenings completed). Initial screening was conducted with a fully automated computerized assessment battery including all outcome measures in the trial. These outcomes later served as the pretreatment scores for the included participants. Additional questions regarding exclusion criteria (suicidality, psychotic symptoms), demographics (age, gender, and education), current treatment, and treatment history were included in the online assessment. Whenever any data regarding the exclusion criteria were found to be unclear, participants were contacted by phone and asked to provide additional information about their psychotic symptoms and suicidal thoughts or behaviors (20.3%, 217 of participants were contacted by telephone to gather this information). The excluded individuals received an explanation as to why they had not been included and, if necessary, advice on how to seek help. Participants who met all inclusion criteria following diagnostic assessment and who provided informed consent were randomly assigned to either the Internet-based treatment or a waiting list control condition. Randomization was based on a computer-generated randomization list. Treatment started right after providing informed consent without any latency for those participants who were assigned to the Internet-based therapy.

All data reported in the trial were collected online and participants were given standardized reminders to complete the assessments using the online assessment system. They completed the outcome measures at pretreatment (initial screening), posttreatment (right after the treatment), and 3-month follow-up. For ethical reasons, participants assigned to the control condition received treatment directly after completing the waiting period. Thus, there are no follow-up results available for the control condition. The Ethics Committee of the University of Leipzig approved the study. Researchers and psychotherapists were not masked to the intervention. See Figure 1 for the progression of study participants.

### Outcomes

Posttraumatic stress symptoms were assessed by the PDS [23], which asks participants to rate on 17 items the occurrence of symptoms of PTSD (intrusions, avoidance, and hyperarousal) in the past 30 days (from 0=never to 3=nearly always). A diagnosis of PTSD is made only when a patient reports the minimum number of symptoms required by *DSM-IV* for each of the symptom clusters. The PDS yields a sum score measuring the overall severity of posttraumatic stress symptoms. Scores of 20 or higher on the PDS indicate moderate to severe symptom severity [23]. Previous research has confirmed the reliability of the Arabic version of the PDS [24]. The Hopkins Symptom Checklist-25 (HSCL-25) [18] was used to assess depression and anxiety. The HSCL-25 comprises 10 items forming an anxiety subscale and 15 items forming a depression subscale with 2 questions relating to somatic symptoms. Both subscales were used in this trial. Scores range from 1 (little) to 4 (very much). A mean score was calculated by adding all scores and dividing by the number of items. Scores above the cutoff of 1.75 indicate clinically significant distress. The HSCL-25 has proved reliable and valid for measuring depression and anxiety in cross-cultural studies [18].

The somatization subscale of the Symptom Checklist-90-Revised (SCL-90-R) [25] was used to assess somatization. This subscale (SCL) of commonly experienced physical symptoms comprises 12 items each rated on a 5-point Likert scale from 0 (not at all) to 4 (extremely). An overall score is formed by calculating the mean score of all items with higher scores indicating severe somatization symptoms during the last week.

Quality of life was assessed with the EUROHIS-QOL [26], an 8-item scale which measures 4 domains of life quality (psychological, physical, social, and environmental), each represented by 2 items. Responses are given on a 5-point Likert scale ranging from 1 (not at all) to 5 (completely). An overall EUROHIS score is formed by calculating the mean score of all 8 items, with higher scores indicating better quality of life. Schmidt et al [26] have reported the measure to show good reliability and validity across a range of countries. The mean time to complete all questionnaires was 45 minutes with a range from 15 to 60 minutes.

### Treatment

#### Internet-Based Treatment

A Dutch Internet-based CBT manual (*Interapy* [15]) was translated into Arabic and culturally adapted for this treatment program. Translations were conducted by different native-speaking psychotherapists following the guidelines for cross-cultural translations [27]. All texts were translated to Modern Standard Arabic because this is the standard for written language in Arab countries and readable for all participants independent from their dialect. The treatment protocol builds on evidence-based principles of CBT for PTSD [28]. Specifically, confrontation with the traumatic event has proven to be an important element of effective psychotherapy for PTSD and has been found to significantly reduce avoidance behavior. The treatment consisted of 2 weekly structured writing activities assigned each week over a period of 5 weeks. There were 3 treatment phases: (1) self-confrontation with the traumatic event, (2) cognitive restructuring, and (3) social sharing.

The basic structure of 10 writing assignments proved to be acceptable in the pilot study [22]. However, based on the evaluation of the pilot study, a number of substantial changes concerning the content of the modules had to be implemented. Patients’ expectations of health care professionals are culturally shaped. Compared to Interapy, this approach uses a more pronounced directive therapeutic stance. In Muslim countries, the health care professional is an authoritative and highly respected figure who gives expert advice. Therefore, straight instructions and responsibility for therapeutic choices are expected. Refusal to give explicit advice and lack of assertion are associated with incompetence and indecisiveness of the therapist and are met with irritation and may even prompt discontinuation of the therapy by a patient.

In the first phase of self-confrontation, the participants were asked to write 4 essays describing the traumatic event and its circumstances in as much detail as possible, in the first person and in the present tense. In contrast to Western trials, participants were explicitly asked not to mentioned specific places or names of persons who were involved due to basic precautionary measures. In the second phase of cognitive restructuring, they had another 4 writing assignments taking the form of a supportive letter to a hypothetical friend who had experienced the same traumatic event. The aim of this phase was to provide new perspectives on the traumatic event. In this module, cultural norms came explicitly into play. Knowledge of the Koran proved extremely helpful. Therapists frequently used quotes and helpful metaphors from the Koran that could inspire patients to take a different perspective and challenge their dysfunctional thoughts.

Generally, the therapists expressed explicit respect toward and appreciation of the concept of the family. However, female participants who had experienced sexual violence were explicitly discouraged from disclosing their traumatic experiences to other family members due to potential serious social consequences of known dishonor (ie, due to experienced sexual violence). The third and final phase of social sharing focused on a symbolic farewell letter (2 assignments) that participants were normally instructed to address to themselves, to a person connected with the traumatic event, or to a friend. In this study, these choices were limited to a letter directed to themselves because of the previously described potentially aversive consequences. All communication with participants was asynchronous. Whenever participants did not conduct their writing assignment, they received a short reminder via email. If no response was received after 2 email reminders, the participant was contacted by phone to encourage them to complete the treatment.

#### Therapists

The participating therapists were 8 native Arabic-speaking psychotherapists or psychiatrists living in Iraq, Palestine, Syria, the Emirates, or Europe. Therapists were trained in 7-day workshops in Europe that focused on the handling of the treatment manual, special features of Internet-based therapy, and how to solve common problems in an online communication setting. After participating in the workshop, the therapists completed an introduction phase with being monitored continuously by a supervisor who read all texts and observed the treatment process. Only after completing this phase successfully did the therapists start to work independently (participating in weekly supervision sessions, either face-to-face or via Skype). The therapist provided individually written feedback and instructions on the next writing assignment within 1 working day. The therapist time involved in responding to texts ranged from 20 to 50 minutes per text, depending on the therapist’s experience with Internet-based therapies.

#### Control Condition

Participants assigned to the control condition were asked to complete a waiting period of 6 weeks. Afterwards, they received the same Internet-based intervention as the treatment group. Because they received treatment straight after completing the waiting period, no relevant follow-up results are available for the control group.

### Statistical Analysis

Statistical analyses were performed with the SPSS version 19.0 for Mac (IBM Corp, Armonk, NY, USA). Data distributions were approximately normal and did not require transformation. As a primary analysis, we performed mixed design ANOVAs with time as the within-subject and condition as the between-subject factors. These analyses were based on an intention-to-treat (ITT) design, including all dropouts to estimate the efficacy of the treatment compared to a waiting list control group. Whenever posttreatment and follow-up scores were not available for a participant, the last observation data were carried forward. Because our repeated-measures variable had only 2 levels, the assumption of sphericity was met and it was not necessary to apply a correction factor to the degrees of freedom [29]. According to Everitt and Howell [29], it is not meaningful to interpret main effects if there are strong interaction effects. Therefore, we abstained from reporting main effects of the ANOVA. In addition to the ITT analysis, we also performed a completer analysis, as proposed by Myers [30]. Chi-square tests and *t* tests were used to determine how similar people who dropped out and people who completed the treatment were, and to assess any differences between the treatment and control groups at baseline. To assess the magnitude of change in mean symptoms between baseline and posttest and between baseline and 3-month follow-up, we calculated effect sizes using Cohen’s *d* for repeated measures. An effect size of *d*=0.80 for a psychological treatment is typically considered large [31]. Moreover, 2 indicators were used to examine whether there was not only a statistical change, but also a clinically significant effect: the reliable change index (RCI) and the clinically significant change following Jacobson and Truax [32]. The RCI is used to determine whether the change observed goes beyond expected measurement fluctuations. The RCI considers measurement error and its effects on variability of scores and is computed by subtracting the subject’s posttest score from his or her pretest score and dividing this value by the standard error of difference between the 2 test scores. Clinically significant change following Jacobson and Truax [32] was determined as scoring below the clinical cutoff (<20 for PDS and <1.75 for the HSCL depression and anxiety subscale).

### Role of the Funding Source

The sponsors of the study had no role in the study design, data collection, data analysis, data interpretation, or writing of the report. The corresponding author had full access to the study data and had responsibility for the decision to submit for publication.

## Results

### Baseline Data

Baseline data of the intrusion (mean 8.32, SD 3.90), avoidance (mean 12.65, SD 3.79), and hyperarousal subscales (mean 9.53, SD 3.32) indicated that all patients suffered from posttraumatic stress (mean 30.50, SD 8.10) and 140 of 159 participants (88.0%) scored above a PDS score of 20.0 indicating moderate to severe symptom levels. Participants also reported high levels of anxiety (mean 2.90, SD 0.60) and depression (mean 3.07, SD 0.54). Table 1 summarizes demographic data and sample characteristics of each group. With the exception of disclosure (χ^2^_1_=4.7, *P*=.02), there were no systematic group differences in any of the sociodemographic variables or in baseline data at pretreatment. Therefore, we did not include any covariates in the analysis.

To investigate differences in outcome for the people who completed the treatment within the planned time frame and those who took a lot longer, we divided the sample into 2 groups (shorter vs longer treatment duration) using a median-split approach. Results indicate that the 2 groups did not differ in their baseline posttraumatic stress (*t*_45_=–1.30, *P*=.20), anxiety (*t*_45_=–1.21, *P*=.23), and depression level (*t*_45_=–1.54, *P*=.13). Similarly, there were no systematic group differences at posttreatment with respect to these variables (posttraumatic stress symptoms: *t*_45_=–0.78, *P*=.44; anxiety: *t*_45_=–1.26, *P*=.22; and depression: *t*_45_=–1.29, *P*=.20).

### Dropout Analysis

In the treatment group, 47 of 79 participants (59%) completed the posttreatment assessment. The treatment group did not differ from the control group in terms of attrition rate (χ^2^_1_=0.0, *P*=.92). The reasons for treatment dropout were often difficult to discern because 20 of 32 participants (63%) did not respond to emails or telephone calls. However, we ascertained that 3 participants were lost due to difficulties with electricity and Internet access. Two patients had to terminate the treatment due to hospitalization and 2 were referred to local psychiatrists. Two participants preferred face-to-face therapy, whereas another 3 patients completed the treatment but not the posttreatment assessment. Completers and noncompleters did not differ with respect to any sociodemographic variables (eg, age: *t*_77_=–0.20, *P*=.84; gender: χ^2^_1_=1.5, *P*=.22; educational level: χ^2^_3_=7.2, *P*=.07; marital status: χ^2^_3_=4.1, *P*=.25; or professional status: χ^2^_2_=3.4, *P*=.19). At baseline, there were no differences in their posttraumatic stress symptoms (*t*_77_=0.68, *P*=.50), anxiety (*t*_77_=0.28, *P*=.78), or depression levels (*t*_77_=–0.27, *P*=.79). Additionally, completers and noncompleters did not differ with respect to the number of traumatic events experienced (*t*_77_=0.64, *P*=.52) or the type of trauma (χ^2^_3_=0.8, *P*=.86).

**Table 1:** Demographic and descriptive characteristics of the treatment and waiting list control groups at baseline (N=159).

Demographic characteristics		Treatment	Control	*t*_157_	χ^2^ (df)	*P*
Traumatic events, mean (SD)		3.67 (3.01)	3.03 (2.61)	1.68		.10
Age (years), mean (SD)		29.11 (8.20)	27.15 (6.48)	1.45		.15
Age (years), range		18-56	18-43			
**Gender, n (%)**					1.0 (1)	.31
	Female	76 (60)	69 (55)			
**Educational level, n (%)**					6.1 (3)	.11
	Completed secondary school	22 (17)	35 (28)			
	University	71 (56)	48 (38)			
**Marital status, n (%)**					2.7 (3)	.44
	Single	68 (53)	56 (45)			
	Partnership/married	26 (20)	37 (30)			
**Professional status, n (%)**					1.3 (2)	.51
	Student	23 (18)	31 (25)			
	Employed	44 (35)	33 (26)			
	Unemployed	33 (26)	36 (29)			
**Type of trauma, n (%)**					0.3 (3)	.96
	Killing of a family member	14 (11)	16 (13)			
	Sexual violence related to war/sexual abuse	39 (31)	40 (32)			
	Violence/war/torture	20 (16)	18 (14)			
	Others (eg, kidnapping, witnessing bomb attacks)	27 (21)	26 (21)			
**Time since trauma (primary traumatic event), n (%)**					4.4 (5)	.50
	Less than 6 months	13 (10)	10 (8)			
	6 months to 3 years	22 (17)	18 (14)			
	More than 3 years	65 (51)	70 (56)			
**Treatment history, n (%)**					0.0 (1)	.96
	Yes	8 (6)	8 (6)			
**Disclosure, n (%)**					4.7 (1)	.02
	Yes	78 (62)	63 (50)			

### Primary Analysis: Intention-to-Treat

Table 2 presents means and standard deviations of baseline, posttreatment, and 3-month follow-up scores for all outcome measures in both experimental conditions. It also presents the group×time interaction of the ANOVAs, whether the group change from pre- to posttreatment is significant, and between-group effect sizes for all outcome measures. As shown, there were significant interaction effects for all outcome variables. This result confirms that the decrease in the primary outcomes intrusion, avoidance, and hyperarousal (PDS) as well as in the secondary outcomes anxiety, depression (HSCL-25), and somatization (SCL) was significantly higher in the treatment group than in the control group. Furthermore participants receiving the Internet-based treatment showed a significant increase in satisfaction with life relative to the control group (*F*_1,157_=44.20, *P*<.001). Despite the high attrition rate of 41% (32/79 in the treatment group), the 3-month follow-up indicated that the results remained stable after treatment. Individuals in the control condition showed no improvement in trauma-related symptoms (PDS subscales: intrusion, avoidance, hyperarousal) or in the secondary outcomes anxiety, depression, somatization, or satisfaction with life. In the treatment group, the ITT analysis revealed moderate to large within-group effect sizes from baseline to posttreatment for the primary outcomes of intrusion (*d*=0.78), avoidance (*d*=0.81), and hyperarousal (*d*=0.86). Large effect sizes were found for the depression (*d*=0.92) and anxiety (*d*=0.84) subscales, and moderate effect sizes for the somatization (*d*=0.42) and quality of life (*d*=0.76) subscales. Furthermore, moderate to large effect sizes (*d*=0.40-0.84) were calculated from baseline to follow-up. In the control condition, all effect sizes were close to zero (*d*=–0.08 to 0.13).

To test for interdependence among dependent variables, we performed a MANOVA for repeated measures (intrusion, avoidance, hyperarousal) with time (pre- and posttreatment) as the within-subject factor and condition as the between-subject factor revealing a significant interaction effect (*F*_1,157_=44.29, *P*<.001). A MANOVA for repeated measures (anxiety and depression) also yielded a significant interaction effect (*F*_1,157_=38.61, *P*<.001).

### Secondary Analysis: Completer

Table 3 presents means and standard deviations of baseline, posttreatment, and follow-up scores for all outcome measures for the subgroup of participants who completed the treatment. It also presents the group×time interaction indicating whether the group change from pre- to posttreatment was significant as well as between-group effect sizes for all outcome measures. As shown, there were significant interaction effects for all outcome variables. As hypothesized, the effect sizes of these factors were larger than in the ITT analysis. With the exception of the secondary outcomes life satisfaction and depression, the completers showed ongoing arithmetical improvement in all outcomes up to the 3-month follow-up. However, these changes did not reach statistical significance. The results of the completer analysis provide further confirmation that both trauma-related symptoms and secondary outcomes, such as anxiety, depression, and somatization, decreased in all patients receiving the treatment. In contrast, there was no significant change from baseline to posttreatment in the control condition. Very large within-group effect sizes were found for posttraumatic stress symptoms (intrusion, avoidance, hyperarousal) from pre- to posttreatment (*d*=1.42-1.53) and 3-month follow-up (*d*=1.95-2.07) in the treatment condition. Large effect sizes were also found for the depression (*d*=1.69) and anxiety (*d*=1.44) subscales, and for quality of life (*d*=1.30) and somatization (*d*=0.68) at posttreatment and follow-up (*d*=1.69, *d*=1.67, *d*=1.09, and *d*=0.83, respectively). Only small effect sizes were found in the control condition (*d*=–0.15 to 0.25).

**Table 2:** Results of mixed design ANOVAs for the treatment and waiting list control groups at baseline, posttreatment, and 3-month follow-up: intention-to-treat analysis.

Outcome measures			Groups×pre-post, mean (SD)		*F*_1,157_	*P*	*d*
			Treatment (n=79)	Control (n=80)			
**PDS**							
	**Intrusion**				30.74	<.001	0.72
		Baseline	8.32^a^ (3.98)	8.33^a^ (3.84)			
		Posttreatment	5.09^b^ (4.33)	8.06^a^ (3.89)			
		Follow-up	5.37^b^ (4.59)	—			
	**Avoidance**				34.26	<.001	0.92
		Baseline	12.49^a^ (3.77)	12.80^a^ (3.82)			
		Posttreatment	8.78^b^ (5.38)	13.04^a^ (3.78)			
		Follow-up	8.97^b^ (5.15)	—			
	**Hyperarousal**				28.58	<.001	0.68
		Baseline	9.54^a^ (3.07)	9.52^a^ (3.58)			
		Posttreatment	6.42^b^ (4.19)	9.07^a^ (3.64)			
		Follow-up	6.53^b^ (4.09)	—			
	**Total score**				44.29	<.001	0.92
		Baseline	30.35^a^ (8.16)	30.65^a^ (8.10)			
		Posttreatment	20.29^b^ (12.45)	30.17^a^ (8.70)			
		Follow-up	20.87^b^ (12.37)	—			
**HSCL-25**							
	**Anxiety**				28.30	<.001	0.79
		Baseline	2.88^a^ (0.60)	2.92^a^ (0.61)			
		Posttreatment	2.29^b^ (0.81)	2.85^a^ (0.61)			
		Follow-up	2.39^b^ (0.76)	—			
	**Depression**				40.66	<.001	1.03
		Baseline	3.04^a^ (0.58)	3.10^a^ (0.50)			
		Posttreatment	2.36^b^ (0.90)	3.11^a^ (0.50)			
		Follow-up	2.54^b^ (0.85)	—			
**SCL: Somatization**					11.68	<.001	0.56
		Baseline	1.44^a^ (0.84)	1.50^a^ (0.90)			
		Posttreatment	1.09^b^ (0.81)	1.56^a^ (0.88)			
		Follow-up	1.11^b^ (0.82)	—			
**EUROHIS: Life satisfaction**					44.20	<.001	0.84
		Baseline	2.33^a^ (0.73)	2.33^a^ (0.79)			
		Posttreatment	2.97^b^ (0.95)	2.27^a^ (0.71)			
		Follow-up	2.72^b^ (0.82)	—			

^a^ Means within column grouping that share this superscript do not differ at *P*=.05.

^b^ Means within column grouping that share this superscript do not differ at *P*=.05.

**Table 3:** Results of mixed design ANOVAs for the treatment and waiting list control groups at baseline, posttreatment, and 3-month follow-up: completer analysis.

Outcome measures			Groups×pre-post, mean (SD)		*F*_1,92_	*P*	η^2^_p_
			Treatment (n=47)^a^	Control (n=47)			
**PDS**							
	**Intrusion**				43.89	<.001	1.50
		Baseline	8.28^b^ (4.09)	8.21^b^ (3.51)			
		Posttreatment	2.85^c^ (3.01)	7.77^b^ (3.56)			
		Follow-up	1.93^c^ (2.42)	—			
	**Avoidance**				45.50	<.001	1.59
		Baseline	12.70^b^ (3.71)	13.13^b^ (3.85)			
		Posttreatment	6.47^c^ (5.04)	13.53^b^ (3.75)			
		Follow-up	5.29^c^ (3.77)	—			
	**Hyperarousal**				42.41	<.001	1.42
		Baseline	9.89^b^ (2.98)	10.47^b^ (2.94)			
		Posttreatment	4.64^c^ (3.87)	9.70^b^ (3.26)			
		Follow-up	3.68^c^ (3.03)	—			
	**Total score**				74.85	<.001	1.77
		Baseline	30.87^b^ (8.13)	31.81^b^ (7.13)			
		Posttreatment	13.96^c^ (10.75)	31.00^b^ (8.36)			
		Follow-up	10.89^c^ (7.91)	—			
**HSCL-25**							
	**Anxiety**				40.71	<.001	1.56
		Baseline	2.90^b^ (0.64)	3.04^b^ (0.51)			
		Posttreatment	1.91^c^ (0.74)	2.91^b^ (0.54)			
		Follow-up	1.85^c^ (0.62)	—			
	**Depression**				59.58	<.001	2.04
		Baseline	3.03^b^ (0.59)	3.16^b^ (0.48)			
		Posttreatment	1.88^c^ (0.77)	3.18^b^ (0.47)			
		Follow-up	1.94^c^ (0.70)	—			
**SCL**							
	**Somatization**				12.40	<.001	0.81
		Baseline	1.45^b^ (0.91)	1.40^b^ (0.84)			
		Posttreatment	0.87^c^ (0.79)	1.52^b^ (0.81)			
		Follow-up	0.75^c^ (0.78)	—			
**EUROHIS**							
	**Life satisfaction**				63.99	<.001	1.52
		Baseline	2.33^b^ (0.76)	2.30^b^ (0.82)			
		Posttreatment	3.39^c^ (0.87)	2.20^b^ (0.68)			
		Follow-up	3.16^c^ (0.76)	—			

^a^ Treatment group reduced to n=28 at 3-month follow-up due to dropout.

^b^ Means within column grouping that share this superscript do not differ at *P*=.05.

^c^ Means within column grouping that share this superscript do not differ at *P*=.05.

### Reliable Change and Clinical Significance

Table 4 presents the percentage of patients showing reliable change at posttreatment (completers’ data). According to this criterion, the proportion of participants showing reliable change in the treatment group was significantly higher than in the control group on all outcome measures. Additionally, we assessed whether treatment led to a change in participants’ diagnostic category. At baseline, 74 of 79 participants (94%) in the treatment group and 75 of 80 patients (94%) in the control group scored at or above the clinical cutoff (20) for posttraumatic stress. At posttreatment the percentage was reduced to 34% (16/47) in the treatment group and to 89% (42/47) in the control group. At the 3-month follow-up, only 14% (4/28) of the treatment group participants scored above the clinical cutoff. With regard to the secondary outcome anxiety, 94% (74/79) in the treatment group and all patients in the control group scored above the cutoff at the beginning of the study (1.75). At posttreatment, this was reduced to 45% (21/47) in the treatment group, whereas 98% (46/47) of the control group still showed above-threshold symptoms. At 3-month follow-up, 13 of 28 participants (46%) in the treatment group showed anxiety symptoms above the cutoff score. Similar results were found on the depression subscale; 96% (76/79) of the treatment and 98% (78/80) of the control group participants scored above the cutoff (1.75) at baseline. All control group patients still suffered from depression symptoms at posttreatment, whereas the percentage was reduced to 40% (19/47) in the treatment group. At 3-month follow-up, this increased to 54% (15/28) of the treatment group participants. Furthermore, 29 of 47 patients (62%) recovered from posttraumatic stress symptoms (reliable change and PDS score<20) in the intervention group compared to 1 (2%) in the control group at posttreatment (OR 74.19, 95% CI 9.93-585.8, *P*<.001), indicating that the chance of recovering was 74.19 times higher in the treatment group than in the control group.

**Table 4:** Percentage of patients showing reliable change at posttreatment.

Outcome measures		Group, n (%)		χ^2^_1_	*P*
		Treatment (n=47)	Control (n=47)		
**PDS**					
	Intrusion	21 (45)	1 (2)	23.7	<.001
	Avoidance	26 (55)	4 (9)	23.7	<.001
	Hyperarousal	27 (57)	7 (15)	18.4	<.001
	Total score	35 (74)	3 (6)	45.2	<.001
**HSCL-25**					
	Anxiety	31 (66)	5 (11)	30.4	<.001
	Depression	35 (74)	4 (9)	42.1	<.001

### Technology Approval und Feasibility

Using the Distress/Endorsement Validation Scale (DEVS [33]) we asked participants about their experience of the treatment. Despite the short-term nature of the intervention, 78% (37/47) of the participants considered the duration to be sufficient; 74% (35/47) spent up to 2 hours a week on the writing therapy. Furthermore, 87% (41/47) of participants regarded the therapy as clearly understandable and as an effective method for reducing tension and exhaustion, 74% (35/47) experienced a marked decrease in their symptoms, and 76% (36/47) would recommend the treatment to others.

## Discussion

The aim of this study was to investigate whether it is possible to produce significant and sustained reduction of posttraumatic stress in participants living in an unstable conflict region using a brief Internet-delivered intervention. We observed significant reductions in posttraumatic stress symptom severity in all symptom clusters, and the effect sizes were of a similar magnitude to those reported for Western samples using the same treatment protocol [15-17,34-39]. In addition, the treatment had significant benefits with respect to symptoms of depression and anxiety and quality of life. Although many of the patients continued to experience difficulties in terms of exposure to life-threatening situations and severe human rights violations during the course of the treatment, they nevertheless benefited psychologically from the intervention.

The attrition rate of 41% (32 of 79 participants in the treatment group and 33 of 80 patients in the control group) was relatively high. A potential explanation for this may be that ongoing violence and economic insecurity keeps patients in a constant state of hyperalertness that calls for a shift of attention to primary day-to-day needs. We tried to determine reasons for dropout by emailing an additional questionnaire, but few people replied. A number of participants questioned the neutrality of the website and the treatment and were concerned that the intervention was supported by a foreign secret service (eg, CIA, Mossad). Others reported technical problems or a lack of privacy at home to write undisturbed by family members. The Dutch Internet-based PTSD study conducted before 2003 [15] also reported a relatively high dropout rate of 43%. An analysis by Lange and colleagues [15] showed that about 59% of the dropouts had stopped the treatment because of technical problems. This is likely to be a key issue in conflict regions, where slow or instable Internet connections or power cuts are still a common problem, and may add to dropout. Despite the attrition rate, the ITT analysis still revealed large effect sizes for posttraumatic stress and depression. Given the high dropout rate, however, ITT analysis may be an overly conservative approach and may have underestimated the effectiveness of this novel approach as indicated by the very large effect sizes of treatment observed in the completer analysis.

A number of limitations demand further comment. First of all, assessment of psychopathology was exclusively based on self-rating questionnaires. A clinical interview would have facilitated more accurate information and clinical diagnosis, and this should be implemented in future trials.

The use of using a waiting list trial design poses substantial limitations on the validity and generalizability of the results. Because the waiting list control condition received treatment after the waiting period, effects in the follow-up intervals can only be estimated based on within-group effect sizes. Furthermore, an active control condition using an alternative evidence-based treatment protocol would have produced more valid data concerning the specific efficacy of this treatment approach. In addition, we found a gender bias because 74% (35 of 47 participants in the treatment group) of completers were female. These figures are comparable with Western treatment samples, but clearly not representative of the general population in this region. Women frequently experience rape or sexual abuse by armed groups in wars and civil conflicts. In Arab countries, women exposed to sexual abuse are often considered to be dishonored; therefore, many of them do not seek help for their psychological problems. Often, they do not risk confiding in others because this leaves them vulnerable to stigma and ostracism and could have life-threatening consequences. The anonymity of the Internet may encourage these women to seek therapeutic treatment. Finally, our sample was very well educated. This is in-line with other Internet-based samples [40,41]. It seems that at present, Internet-based interventions do not generally manage to engage less well-educated people in an intervention [42], independent of the type of program and country of origin.

Future research should expand treatment delivery techniques, especially focusing on conflict regions. New developments in the context of communication technology report an increasing use of mobile apps (ie, mobile phones) in the treatment of mental health problems. As mobile phone usage in the Middle East is growing, apps could be an additional way to extend treatment accessibility.

Finally, PTSD is only 1 outcome of chronic exposure to traumatic events in postconflict countries. In their extensive meta-analysis of 181 studies on psychological consequences of war and deportation, Steele and colleagues [43] found that rates of depression (30.8%) match those for PTSD (30.6%). A randomized controlled trial testing an Internet-based CBT treatment for depression in Arabic postconflict countries is currently underway.

To our knowledge, this is the first randomized controlled trial of any form of psychological intervention for people in the Middle East suffering from PTSD. The written narratives produced by our participants impressively document the toll of decades of dictatorship and war. Seventy psychiatrists are currently registered in Iraq, which has a population of 30 million. It will take decades before local services are able to provide sufficient health care for the country’s citizens. Our findings show, even when living in difficult conditions, people with posttraumatic stress symptoms benefit from a CBT provided entirely through the Internet. Findings on the program’s uptake and applicability confirm that new technologies can be used to provide humanitarian aid in the form of e-mental health services, even in areas that remain highly unstable. The opportunity to disseminate evidence-based, accessible interventions in regions where human rights violations are common represents an important contribution to humanitarian aid and has substantial public health implications.

## Multimedia Appendix 1

CONSORT-EHEALTH checklist V1.6.2 [44].

## Abbreviations

CBT: cognitive behavioral therapy
DEVS: Distress/Endorsement Validation Scale
DSM-IV: Diagnostic and Statistical Manual of Mental Disorders (4th Edition)
HSCL-25: Hopkins Symptom Checklist-25
ITT: intention-to-treat
PDS: Posttraumatic Stress Diagnostic Scale
PTSD: posttraumatic stress disorder
RCI: reliable change index
SCL-90-R: Symptom Checklist-90-Revised
