# Supplementary material for: Web-Based Psychotherapy for Posttraumatic Stress Disorder in War-Traumatized Arab Patients: Randomized Controlled Trial
Source: J Med Internet Res. 2015 Mar 20;17(3):e71. doi: 10.2196/jmir.3582 (PMC4385175; doi:10.2196/jmir.3582)
Supplement: Supplementary file 1 [file jmir_v17i3e71_app1.pdf]

CONSORT-EHEALTH Checklist V1.6.2 Report

(based on CONSORT-EHEALTH V1.6), available at [http://tinyurl.com/consort-ehealth-v1-6].

3582

Date completed

9/25/2014 9:25:38

by

Christine Knaevelsrud

"Web-based psychotherapy for posttraumatic stress disorder in war-traumatized Arab patients: a parallel group randomized controlled trial"

TITLE

1a-i) Identify the mode of delivery in the title

"Web-based psychotherapy for posttraumatic stress disorder in war-traumatized Arab patients: a parallel group randomized controlled trial"

1a-ii) Non-web-based components or important co-interventions in title

"Web-based psychotherapy for posttraumatic stress disorder in war-traumatized Arab patients: a parallel group randomized controlled trial"

1a-iii) Primary condition or target group in the title

"Web-based psychotherapy for posttraumatic stress disorder in war-traumatized Arab patients: a parallel group randomized controlled trial"

ABSTRACT

1b-i) Key features/functionalities/components of the intervention and comparator in the METHODS section of the ABSTRACT

"Methods
A total of 159 individuals with posttraumatic stress disorder participated in a parallel-group randomized trial. Participants were randomly allocated by a computer-generated sequence to a treatment group (n = 79) or a waiting list control group (n = 80). The treatment group received two weekly 45-minute cognitive-behavioral interventions via Internet over a 5-week period (10 sessions in total). The primary outcome was recovery from posttraumatic stress symptoms."

1b-ii) Level of human involvement in the METHODS section of the ABSTRACT

1b-iii) Open vs. closed, web-based (self-assessment) vs. face-to-face assessments in the METHODS section of the ABSTRACT

"Recruitment for this randomized controlled trial took place from January 2009 to November 2011. Participants were recruited through radio, TV, and newspaper announcements, as well as health-related websites, specifically in Iraq. Information about the study was published regularly on a Facebook page."

1b-iv) RESULTS section in abstract must contain use data

"A total of 159 individuals with posttraumatic stress disorder participated in a parallel-group randomized trial. Participants were randomly allocated by a computer-generated sequence to a treatment group (n = 79) or a waiting list control group (n = 80). The treatment group received two weekly 45-minute cognitive-behavioral interventions via Internet over a 5-week period (10 sessions in total). The primary outcome was recovery from posttraumatic stress symptoms.
Results
Posttraumatic stress symptoms were significantly reduced from baseline to posttreatment (intent-to-treat analysis) in the treatment group relative to the control group ($F(1,157) = 44.29$, $p < .001$, $d = 0.92$). Treatment effects were sustained at 3-month follow-up. Completer analysis indicated that 29 of 47 patients (62%) in the treatment group had recovered from posttraumatic stress symptoms at posttreatment (reliable change and PDS score < 20) versus 1 patient (2%) in the control group (OR 74.19, 95% CI [9.93–585.8], $p < .001$) indicating that the chance of recovering was 74.19 times higher in the treatment than in the control group."

1b-v) CONCLUSIONS/DISCUSSION in abstract for negative trials

No negative results

INTRODUCTION

2a-i) Problem and the type of system/solution

"Internet-based approaches may provide a unique treatment alternative in conflict areas where there is an urgent need for psychological care that is easily
accessible, independent of the location of the therapist, and relatively inexpensive. "

2a-ii) Scientific background, rationale: What is known about the (type of) system

"Internet-based delivery of psychotherapeutic interventions has become increasingly established in the Western world. In particular, interventions developed for patients with PTSD have been shown to produce significant reductions in PTSD symptoms as well as in associated psychopathology such as depression and anxiety [12-17]."

METHODS

3a) CONSORT: Description of trial design (such as parallel, factorial) including allocation ratio

"We hypothesized that the Internet-based treatment would produce a significantly greater improvement on the outcome compared to the control condition. Additionally, participants in the treatment group were assessed at a 3-month follow-up session to investigate the maintenance of the treatment effects. In the control group no follow-up assessments were conducted as all of the control group participants received treatment after completing their waiting period."

3b) CONSORT: Important changes to methods after trial commencement (such as eligibility criteria), with reasons

No changes

3b-i) Bug fixes, Downtimes, Content Changes

It was an already proven system which was extensively tested in a previous pilot study

4a) CONSORT: Eligibility criteria for participants

"To be included in the study, participants had to have a history of trauma according to the DSM-IV criteria accompanied by posttraumatic stress symptoms, knowledge of Arabic, and age between 18 and 65 years. The Posttraumatic Stress Diagnostic Scale (PDS) was used to watch out if patients report the minimum number of symptoms required by DSM-IV for each of the symptom clusters (at least one intrusion, three avoidance and two hyperarousal symptoms). Additionally the minimum score on the PDS to be included in the trial was 11 indicating moderate symptom severity. Applicants were excluded if they met one of the following criteria: currently receiving treatment elsewhere, substance abuse or dependence, high risk of suicide, psychotic symptoms, and low symptom severity. "

4a-i) Computer / Internet literacy

4a-ii) Open vs. closed, web-based vs. face-to-face assessments:

Initial screening was conducted with a fully automated computerized assessment battery including all outcome measures in the trial. These outcomes later served as the pretreatment scores for the included participants. Additional questions regarding exclusion criteria (suicidality, psychotic symptoms), demographics (age, gender and education), current treatment and treatment history were included in the online assessment. Whenever any data regarding the exclusion criteria were found to be unclear, participants were contacted by phone and asked to provide additional information about their psychotic symptoms and suicidal thoughts or behaviors (20% of participants have been contacted by telephone to gather these information)."

4a-iii) Information giving during recruitment

"The study website (www.ilajnafsy.org) provided general information about PTSD, online assessment, and the treatment program. Potential participants were informed about the study and received information about (a) posttraumatic stress reactions, (b) the study and its inclusion and exclusion criteria, (c) the Internet-based treatment, and (d) other treatment alternatives. A detailed description of the three treatment modules and the text-based form of the intervention was also given to the participants along with the patient information."

4b) CONSORT: Settings and locations where the data were collected

"The study was carried out in Berlin (Treatment Center for Torture Victims/ Freie University Berlin). Recruitment for this randomized controlled trial took place from January 2009 to November 2011."

4b-i) Report if outcomes were (self-)assessed through online questionnaires

Potential patients logged in and completed the screening questionnaires online (1070 screenings completed). Initial screening was conducted with a fully automated computerized assessment battery including all outcome measures in the trial. These outcomes later served as the pretreatment scores for the included participants. Additional questions regarding exclusion criteria (suicidality, psychotic symptoms), demographics (age, gender and education), current treatment and treatment history were included in the online assessment. Whenever any data regarding the exclusion criteria were found to be unclear, participants were contacted by phone and asked to provide additional information about their psychotic symptoms and suicidal thoughts or behaviors (20% of participants have been contacted by telephone to gather these information)."

4b-ii) Report how institutional affiliations are displayed

5) CONSORT: Describe the interventions for each group with sufficient details to allow replication, including how and when they were actually administered

5-i) Mention names, credential, affiliations of the developers, sponsors, and owners

5-ii) Describe the history/development process

5-iii) Revisions and updating

5-iv) Quality assurance methods

5-v) Ensure replicability by publishing the source code, and/or providing screenshots/screen-capture video, and/or providing flowcharts of the algorithms used

5-vi) Digital preservation

5-vii) Access

Participants did not pay/or received any financial compensation

5-viii) Mode of delivery, features/functionalities/components of the intervention and comparator, and the theoretical framework

Internet-based ttreatment: A Dutch Internet-based cognitive-behavioral approach ttreatment manual (called Interapy; [15]) was translated into Arabic and culturally adapted for the present treatment program. Translations were conducted by different native-speaking psychotherapists following the guidelines for cross-cultural translations [26]). All texts have been translated to Modern Standard Arabic as this is the standard for written language in Arab countries and readable for all participants independent from their dialect. The treatment protocol builds upon evidence-based principles of cognitive-behavioral therapy for PTSD [27]. Specifically, confrontation with the traumatic event has proven to be an important element of effective psychotherapy for PTSD, which has been found to significantly reduce avoidance behavior. The treatment consisted of two weekly structured writing activities assigned each week over a period of five weeks. There were three treatment phases: (a) self-confrontation with the traumatic event, (b) cognitive restructuring, and (c) social sharing.

The basic structure of ten writing assignments proved to be acceptable in the pilot study [21]. However, based on the evaluation of the pilot study a number of substantial changes concerning the content of the modules had to be implemented. Patients' expectations of health care professionals are culturally shaped. Compared to Interapy, this current approach uses a more pronounced directive therapeutic stance. In Muslim countries the health care professional is an authoritative and highly respected figure who gives expert advice. Therefore straight instructions and responsibility for therapeutic choices are expected. Refusal to give explicit advice and lack of assertion are associated with incompetence and indecisiveness of the therapist and are met with irritation and may even prompt discontinuation of the therapy by a patient.

In the first phase of self-confrontation, the participants were asked to write four essays describing the traumatic event and its circumstances in as much detail as possible, in the first person and in the present tense. In contrast to Western trials, participants were explicitly asked not to mentioned specific places or names of persons who were involved, due to basic precautionary measures. In the second phase of cognitive restructuring, they had another four writing assignments, taking the form of a supportive letter to a hypothetical friend who had experienced the same traumatic event. The aim of this phase was to provide new perspectives on the traumatic event. In this module, cultural norms came explicitly into play. Knowledge of the Koran proved extremely helpful. Therapists frequently used quotes and helpful metaphors from the Koran that could inspire patients to take a different perspective and challenge their dysfunctional thoughts.

Generally the therapists expressed explicit respect towards and appreciation of the concept of the family. However, female participants who had experienced sexual violence were explicitly discouraged from disclosing their traumatic experiences to other family members due to potential serious social consequences of known dishonor (i.e. due to experienced sexual violence). The third and final phase of social sharing focused on a symbolic farewell letter (two assignments) that participants are normally instructed to address to themselves, to a person connected with the traumatic event, or to a friend. In the current trial these choices were limited to a letter directed to themselves, due to the above described potentially aversive consequences. All communication with participants was asynchronous. Whenever participants did not conduct their writing assignment they received a short reminder via email. If no response was received after two e-mail reminders, the participant was contacted by phone to encourage them to complete the treatment.

Therapists: The participating therapists were 8 native Arabic-speaking psychotherapists or psychiatrists living in Iraq, Palestine, Syria, the Emirates, or Europe. Therapists were trained in 7-day workshops in Europe that focused on the handling of the treatment manual, special features of Internet-based therapy and how to solve common problems in an online communication setting. After participating in the workshop the therapists completed an introduction phase with being monitored continuously by a supervisor who read all texts and observed the treatment process. Only when completing this phase successfully the therapists started to work independent (participating in weekly supervision sessions, either face-to-face or via Skype). The therapist provided individually written feedback and instructions on the next writing assignment within one working day. The therapist time involved in responding to texts ranged from 20 to 50 minutes per text, depending on the therapist's experience with Internet-based therapies.

Control Condition: Participants assigned to the control condition were asked to complete a waiting period of 6 weeks. Afterwards they received the same Internet-based intervention as the treatment group. As they received treatment straight after completing the waiting period, no relevant follow-up results are available for the control group."

5-ix) Describe use parameters

5-x) Clarify the level of human involvement

5-xi) Report any prompts/reminders used

"All communication with participants was asynchronous. Whenever participants did not conduct their writing assignment they received a short reminder via email. If no response was received after two e-mail reminders, the participant was contacted by phone to encourage them to complete the treatment."

5-xii) Describe any co-interventions (incl. training/support)

Therapists were trained in 7-day workshops in Europe that focused on the handling of the treatment manual, special features of Internet-based therapy and how to solve common problems in an online communication setting. After participating in the workshop the therapists completed an introduction phase with being monitored continuously by a supervisor who read all texts and observed the treatment process. Only when completing this phase successfully the therapists started to work independent (participating in weekly supervision sessions, either face-to-face or via Skype)."

6a) CONSORT: Completely defined pre-specified primary and secondary outcome measures, including how and when they were assessed

Assessments were completed at three time points (pre, post, and 3-month follow-up). PTSD:primary outcome; Depression, anxiety, somatization and quality of life: scondary outcome: all self-report

6a-i) Online questionnaires: describe if they were validated for online use and apply CHERRIES items to describe how the questionnaires were designed/deployed

6a-ii) Describe whether and how "use" (including intensity of use/dosage) was defined/measured/monitored

6a-iii) Describe whether, how, and when qualitative feedback from participants was obtained

6b) CONSORT: Any changes to trial outcomes after the trial commenced, with reasons

No changes after study start

7a) CONSORT: How sample size was determined

7a-i) Describe whether and how expected attrition was taken into account when calculating the sample size

7b) CONSORT: When applicable, explanation of any interim analyses and stopping guidelines

No interim analyses

8a) CONSORT: Method used to generate the random allocation sequence

Randomization was based on a computer generated randomization list.

8b) CONSORT: Type of randomisation; details of any restriction (such as blocking and block size)

Unrestricted randomization

9) CONSORT: Mechanism used to implement the random allocation sequence (such as sequentially numbered containers), describing any steps taken to conceal the sequence until interventions were assigned

As there were no restriction applicable a simple computerbased allocation sequence was used

10) CONSORT: Who generated the random allocation sequence, who enrolled participants, and who assigned participants to interventions

Randomization list was accessible only by the two study coordinators. When a new patient enrolled the treatment coordinator contacted the study coordinator and received information on assignment according to the randomization list.

11a) CONSORT: Blinding - If done, who was blinded after assignment to interventions (for example, participants, care providers, those assessing outcomes) and how

11a-i) Specify who was blinded, and who wasn't

No blinding occured.

11a-ii) Discuss e.g., whether participants knew which intervention was the "intervention of interest" and which one was the "comparator"

11b) CONSORT: If relevant, description of the similarity of interventions

not relevant

12a) CONSORT: Statistical methods used to compare groups for primary and secondary outcomes

"Statistical analyses were performed with the Statistic Package for Social Sciences (SPSS), version 19.0 for MAC.
Data distributions were approximately normal and did not require transformation. As a primary analysis we performed mixed design ANOVAs with time as within-subject and condition as between-subject factor. These analyses were based on an intent-to-treat design, including all dropouts to estimate the efficacy of the treatment compared to a waiting list control group. Whenever post-treatment and follow-up scores were not available for a participant, the last observation data were carried forward. Because our repeated-measures variable had only two levels, the assumption of sphericity was met and it was not necessary to apply a correction factor to the degrees of freedom [28]. According to Everitt and Howell [28], it is not meaningful to interpret main effects if there are strong interaction effects. Therefore we abstained from reporting main effects of the ANOVA. In addition to the intent-to-treat analysis, we also performed a completer analysis, as proposed by [29]. Chi-square tests and t-tests were used to determine how similar people who dropped out and people who completed the treatment were, as well as to assess any differences between treatment and control group at baseline."

12a-i) Imputation techniques to deal with attrition / missing values

"Analyses were based on an intent-to-treat design, including all dropouts to estimate the efficacy of the treatment compared to a waiting list control group. Whenever post-treatment and follow-up scores were not available for a participant, the last observation data were carried forward."

12b) CONSORT: Methods for additional analyses, such as subgroup analyses and adjusted analyses

"To assess the magnitude of change in mean symptoms between baseline and posttest and between baseline and 3-month follow-up, we calculated effect sizes using Cohen's d for repeated measures. An effect size of d = 0.80 for a psychological treatment is typically considered large [30]. Moreover, two indicators were used to examine whether there was not only a statistical change, but also a clinically significant effect: the reliable change index (RCI) and the clinically significant change following Jacobson and Truax [31]. The RCI is used to determine whether the change observed goes beyond expected measurement fluctuations. The RCI considers measurement error and its effects on variability of scores and is computed using the formula: RC = x2 – x1 / Sdiff. The subject's pretest score is subtracted from his/her posttest score and divided by the standard error of difference between the two test scores. Clinically significant change following Jacobson and Truax [31] was determined as scoring below the clinical cutoff (< 20 for PDS and < 1.75 for the HSCL depression and anxiety subscale)."

RESULTS

13a) CONSORT: For each group, the numbers of participants who were randomly assigned, received intended treatment, and were analysed for the primary outcome

yes see table 3 in the manuscript

13b) CONSORT: For each group, losses and exclusions after randomisation, together with reasons

yes see flow diagram (figure 1 in the manuscript)

13b-i) Attrition diagram

14a) CONSORT: Dates defining the periods of recruitment and follow-up

Recruitment for this randomized controlled trial took place from January 2009 to November 2011. Follow-up period: 3 months

14a-i) Indicate if critical "secular events" fell into the study period

14b) CONSORT: Why the trial ended or was stopped (early)

not applicable

15) CONSORT: A table showing baseline demographic and clinical characteristics for each group

Table 1

15-i) Report demographics associated with digital divide issues

Table 1

16a) CONSORT: For each group, number of participants (denominator) included in each analysis and whether the analysis was by original assigned groups

16-i) Report multiple "denominators" and provide definitions

Information is correctly provided in table 1-4 of the manuscript

16-ii) Primary analysis should be intent-to-treat

see above - ITT was used

17a) CONSORT: For each primary and secondary outcome, results for each group, and the estimated effect size and its precision (such as 95% confidence interval)

"Posttraumatic stress symptoms were significantly reduced from baseline to posttreatment (intent-to-treat analysis) in the treatment group relative to the control group ($F(1,157) = 44.29$, $p < .001$, $d = 0.92$). Treatment effects were sustained at 3-month follow-up. Completer analysis indicated that 29 of 47 patients (62%) in the treatment group had recovered from posttraumatic stress symptoms at posttreatment (reliable change and PDS score < 20) versus 1 patient (2%) in the control group (OR 74.19, 95% CI [9.93–585.8], $p < .001$) indicating that the chance of recovering was 74.19 times higher in the treatment than in the control group."

17a-i) Presentation of process outcomes such as metrics of use and intensity of use

17b) CONSORT: For binary outcomes, presentation of both absolute and relative effect sizes is recommended

No binary outcomes are reported. Except for clinical significant change - in that case we provided data on absolute numbers and percentages

18) CONSORT: Results of any other analyses performed, including subgroup analyses and adjusted analyses, distinguishing pre-specified from exploratory

No additional subgroup analyses

18-i) Subgroup analysis of comparing only users

19) CONSORT: All important harms or unintended effects in each group

No unintended effects

19-i) Include privacy breaches, technical problems

19-ii) Include qualitative feedback from participants or observations from staff/researchers

DISCUSSION

20) CONSORT: Trial limitations, addressing sources of potential bias, imprecision, multiplicity of analyses

20-i) Typical limitations in ehealth trials

"A number of limitations demand further comment. First of all, assessment of psychopathology was exclusively based on self-rating questionnaires. A clinical interview would have facilitated more accurate information and clinical diagnosis and should be implemented in future trials.
The use of using a waiting-list trial design poses substantial limitations on the validity and generalizability of the results. As the waiting-list control condition received treatment after the waiting period, effects in the follow-up intervals can only be estimated based on within-group effect sizes. Furthermore, an active control condition using an alternative evidence-based treatment protocol would have produced more valid data concerning the specific efficacy of this treatment approach. In addition, we found a gender-bias, as 74% (35 out of 47 participants in the treatment group) of completers were female. These figures are comparable with Western treatment samples, but clearly not representative of the general population in this region. Women frequently experience rape or sexual abuse by armed groups in wars and civil conflicts. Because, in Arab countries, women exposed to sexual abuse are often considered to be dishonored, many of them do not seek help for their psychological problems. Often, they do not risk confiding in others, as this leaves them vulnerable to stigma and ostracism and could have life-threatening consequences. The anonymity of the Internet may encourage these women to seek therapeutic treatment. Finally, our sample was very well educated. This is in line with other internet-based samples [39,40]. It seems that at present, Internet-based interventions do not generally manage to engage less well-educated people in an intervention [41], independent of the type of program and country of origin."

21) CONSORT: Generalisability (external validity, applicability) of the trial findings

21-i) Generalizability to other populations

21-ii) Discuss if there were elements in the RCT that would be different in a routine application setting

22) CONSORT: Interpretation consistent with results, balancing benefits and harms, and considering other relevant evidence

22-i) Restate study questions and summarize the answers suggested by the data, starting with primary outcomes and process outcomes (use)

Information is given as proposed in the discussion

22-ii) Highlight unanswered new questions, suggest future research

Other information

23) CONSORT: Registration number and name of trial registry

The trial is registered with Australian New Zealand Clinical Trial Registry, ACTRN12611001019998.

24) CONSORT: Where the full trial protocol can be accessed, if available

Full trial protocol can be accessed on request at the first author

25) CONSORT: Sources of funding and other support (such as supply of drugs), role of funders

FUNDING:
German Foreign Ministry; Misereor e.V., Germany

X26-i) Comment on ethics committee approval

x26-ii) Outline informed consent procedures

X26-iii) Safety and security procedures

X27-i) State the relation of the study team towards the system being evaluated
